# Evaluation of Splicing on X-box Binding Protein Transcript in Tissue Samples of Colorectal Cancer

**DOI:** 10.7759/cureus.4500

**Published:** 2019-04-19

**Authors:** Azam Ahmadi, Reza Aghabozorgi, Ali Arash Anoushirvani, Mohamadreza Bayatiani, Mohammad Arjomandzadegan, Saeedeh Eslah, Yasaman Sobhani

**Affiliations:** 1 Genetics, Arak University of Medical Sciences, Arak, IRN; 2 Internal Medicine, Arak University of Medical Sciences, Arak, IRN; 3 Radiotherapy and Medical Physics, Arak University of Medical Sciences, Arak, IRN; 4 Miscellaneous, Arak University of Medical Sciences, Arak, IRN; 5 Radiotherapy and Medical Physics, Arak University of Medical Sciences, Arak, IRN

**Keywords:** crc, xbp, splicing

## Abstract

Background

The genetic etiology of colorectal cancer (CRC) is the occurrence of mutation in the genes involved in signal transduction pathways including that of cellular responses to endoplasmic reticulum (ER) stress. This study examines alterations of pre-messenger ribonucleic acid (pre-mRNA) splicing in X-box binding protein (XBP) transcripts related to the ER stress pathway in CRC.

Materials and methods

In this study, samples were deparaffinized and underwent RNA extraction. A total of 30 synthesized complementary deoxyribonucleic acid (cDNA) templates from the extracted RNAs related to tumor and non-tumor CRC samples, collected over three years and containing pathological data, were subjected to semi-quantitative reverse transcriptase polymerase chain reaction (sqRT-PCR). These cDNA templates were amplified in reaction tubes with specific primers for both spliced and non-spliced isoforms of XBP. Results with P< .05 were considered statistically significant.

Results

Microscopic assessment represented lymphocyte-rich effusion in tumor samples. sqRT-PCR electrophoresis results showed spliced and non-spliced forms of XBP messenger RNA in the studied samples. In addition, our data showed there were more than 7.8 times the total number of spliced variants in the marginal tumor samples than in the tumor tissue samples (P<.05).

Conclusion

Alterations of expression in genes involved in stress signaling pathways in cancer have been identified previously. Our results showed an inverse relationship between XBP splicing and CRC tumor tissue, possibly lead to the inactivation of apoptosis in the downstream response to ER stress. However, we propose that the remaining genes in this pathway should undergo gene expression analysis using a greater number of samples.

## Introduction

Colorectal cancer (CRC) is the third most common cancer, with an incidence of 1.36 million people per year worldwide. The genetic causes of this heterogeneous disease are the occurrence of changes in pathway genes including tumor protein p53 (TP53), transforming growth factor beta 1 (TGFB), Janus kinase/ signal transducer and activator of transcription (JAK / STAT), phosphatidylinositol-4,5-bisphosphate 3-kinase (PI3K), and Wnt family member 1 (WNT1) [1-2]. One pathway that plays a role in CRC is the endoplasmic reticulum (ER) stress pathway [3] involved in the stages of development, tissue homeostasis, and key biological processes. This pathway has evolved in eukaryotic cells and is involved in several diseases including neurodegenerative diseases and cancer [4]. Cancer cells are often exposed to hypoxia, hunger, oxidative alteration, and other changes that can lead to ER stress that activates the responsive pathway. Several studies suggested that activation of this pathway can have a paradoxical role in both tumor-promoting and tumor-suppressing activity [5]. Although chemotherapy, radiotherapy, and surgery are used to treat the most common types of cancer, diagnosing cancer at the earliest stages is the current challenge of cancer research. Targeted therapy drugs that target the genes K-ras (KRAS) proto-oncogene, GTPase (KRAS), B-Raf proto-oncogene serine/threonine kinase (BRAF), and SMAD family member 4 (SMAD4) are used with chemotherapy regimens to treat CRC. Some studies recently suggested manipulating components of the ER stress response pathway as a potential therapy for CRC [6].

One of the major functions of the ER network is to coordinate between synthesis and secretion of proteins. The first step of maturation and folding of the secretory and membrane proteins occurs in the ER. Changes in homeostasis lead to the accumulation of unfolded or inappropriately folding proteins, ER stress, and activation of the ER stress response pathway. Depending on the duration and amount of ER stress, the pathway can either send survival signals by activating anti-apoptotic pathways or trigger death signals by inducing programmed cell death [5]. Some cancer drugs that target components of this pathway are at different stages of clinical trials today [7]. Upon ER stress, three transducers (pancreatic eIF-2alpha kinase (PERK), inositol-requiring enzyme-1 (IRE-1), and activating transcription factor 6 (ATF-6)) and a regulator called heat shock protein family A (Hsp70) member 5 (HSPA5) are activated [8].

ATF6 is activated after dissociation from HSPA5 and digested with peptidase in the Golgi apparatus. This portion is transferred into the nucleus as a transcription factor, leading to the expression of some chaperone proteins and X-box Binding Protein (XBP). XBP is transferred into the cytoplasm and spliced by IRE1. The spliced form is also transferred as a transcription factor to the nucleus. If the stress persists, the genes involved in apoptosis are activated. A study of the detailed processes of each of the mentioned components in this signaling pathway is important to understand its molecular mechanisms. XBP is essential for the survival of a tumor under hypoxic conditions [9]. XBP messenger RNA (mRNA) is spliced by IRE1; thus, inhibition of IRE1 will lead to inactivation of XBP splicing [10].

The ER stress response pathway in CRC is very important, and the mechanisms of this pathway in CRC are poorly understood; therefore, we assessed XBP transcript splicing in CRC tumor samples compared to non-tumor or marginal tumor samples. The results of this study may clarify the paradoxical mechanisms of this signaling pathway in CRC.

## Materials and methods

Clinical samples

A total of 30 paraffin-embedded tissue blocks from tissue samples were collected from the pathology department of hospitals in Arak, Iran over the course of three years. Tumor and non-tumor samples of these blocks were confirmed using pathological examination. From each tissue block, one slide was stained with hematoxylin and eosin.

Ribonucleic acid extraction

The collected samples underwent deparaffinization and rehydration, then were digested with Proteinase K. These experiments were performed by adding xylene to tissue then washing with various dilutions of ethanol. After digestion with Proteinase K, RNA extraction was performed according to its protocol (SinaClon, Iran) and the samples were treated with DNaseI (SinaClon, Iran).

Complementary deoxyribonucleic acid synthesis

A two-step cDNA synthesis protocol was performed with the help of Random Hexamer primer and Reverse Transcriptase Enzyme (Cinagen-PR911658) in reaction tubes at a volume of 20 μL according to the optimized protocol for cDNA. The cDNAs were transferred to a -20°C freezer.

Semi-quantitative reverse transcriptase polymerase chain reaction 

sqRT-PCR was optimized by synthesizing cDNA as templates (2 μl) and 2X MasterMix (YTA, Iran) with specific primers (10 pmol) for amplification of spliced and non-spliced variants of XBP with a touchdown program (annealing temperature, 52°C to 62°C) in a thermocycler machine. Used primers amplified a 152 base pair (bp) (non-spliced isoform) or a 126 bp fragment (spliced isoform). The products were loaded on 1.3% agarose gel (Genefanavaran, Iran) which was stained with Safe Stain (SinaClon, Iran). The bands were analyzed using the Gel Doc system (Quantum ST4, Germany). To confirm the amplified products, some of the PCR products were sent to Macrogen (South Korea) for sequencing on an Applied Biosystems (ABI System, 3730xl instrument). The sequence of these primers [10] is presented in Table 1.

**Table 1:** The primers used in this study F xbpSU, forward x-box binding protein spliced and un-spliced, R xbpSU, reverse x-box binding protein spliced and un-spliced

ID	Sequence (5'-3')	Length of oligonucleotide	Tm
F xbpSU	CCTGGTTGCTGAAGAGGAGG	20	55.9
R xbpSU	CCATGGGGAGATGTTCTGGAG	21	56.3
total xbp-F	GCAAGCGACAGCGCCT	16	51.1
total xbp-R	TTTTCAGTTTCCTCCTCAGCG	21	52.4

Statistical analysis

Statistical analysis was performed using MedCalc and GraphPad Prism software version 7.0 (unpaired t-test).

## Results

**Figure 1:**
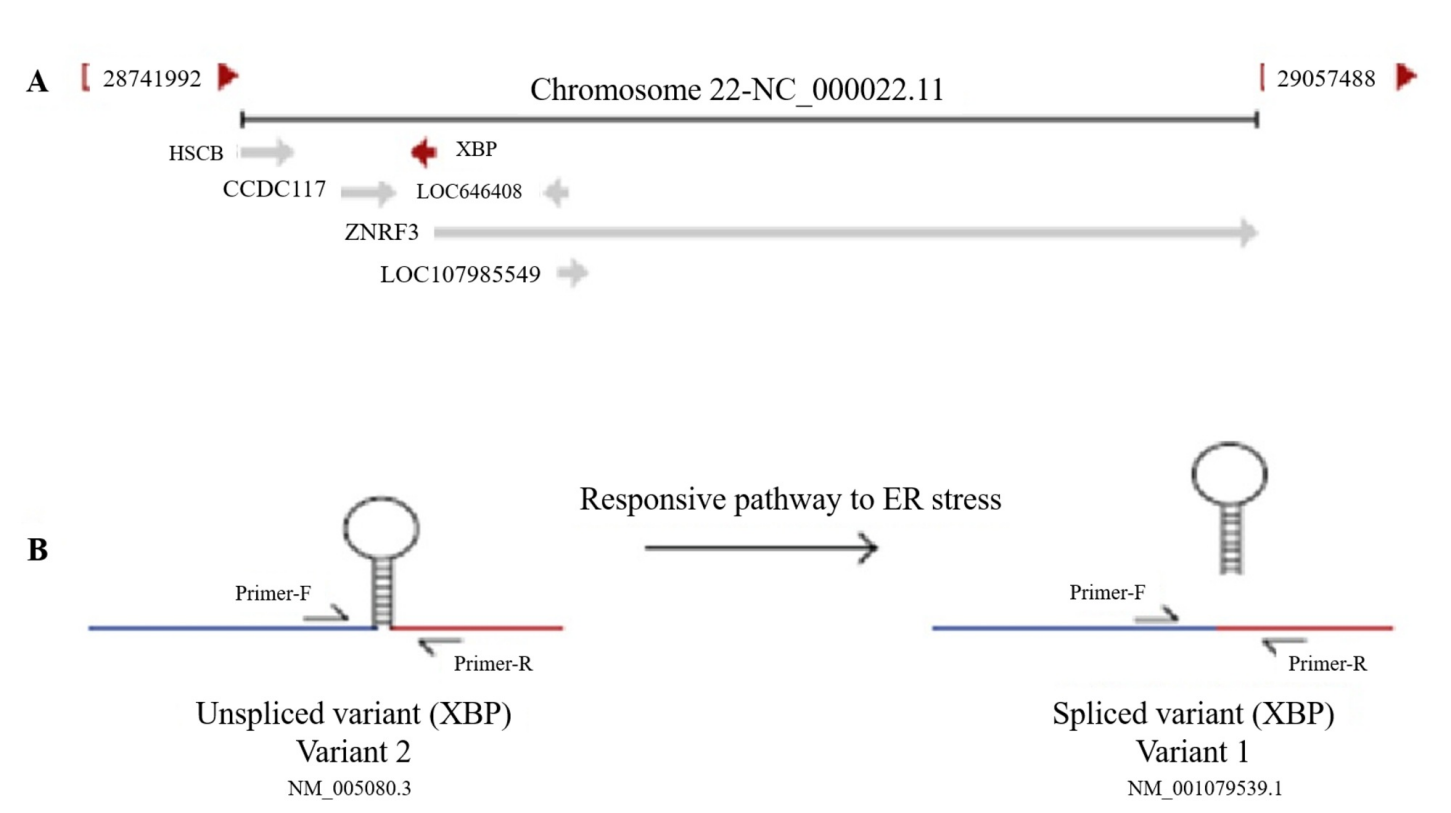
A: Position of XBP gene on chromosome 22, B: Schematic view of XBP splicing XBP: X-box binding protein; ER: endoplasmic reticulum

Bioinformatics analysis determined the XBP gene contains five exons that code several types of transcripts. The type I variant is a longer transcript, although it produces a shorter protein isoform. The type II variant in the coding sequence is shorter than variant 1, resulting in a spliced-type with a C-terminal domain longer than the non-spliced isoform (Figure 1).

The type II variant (spliced) acts as a transcription factor in the pathway responding to ER stress.

**Figure 2:**
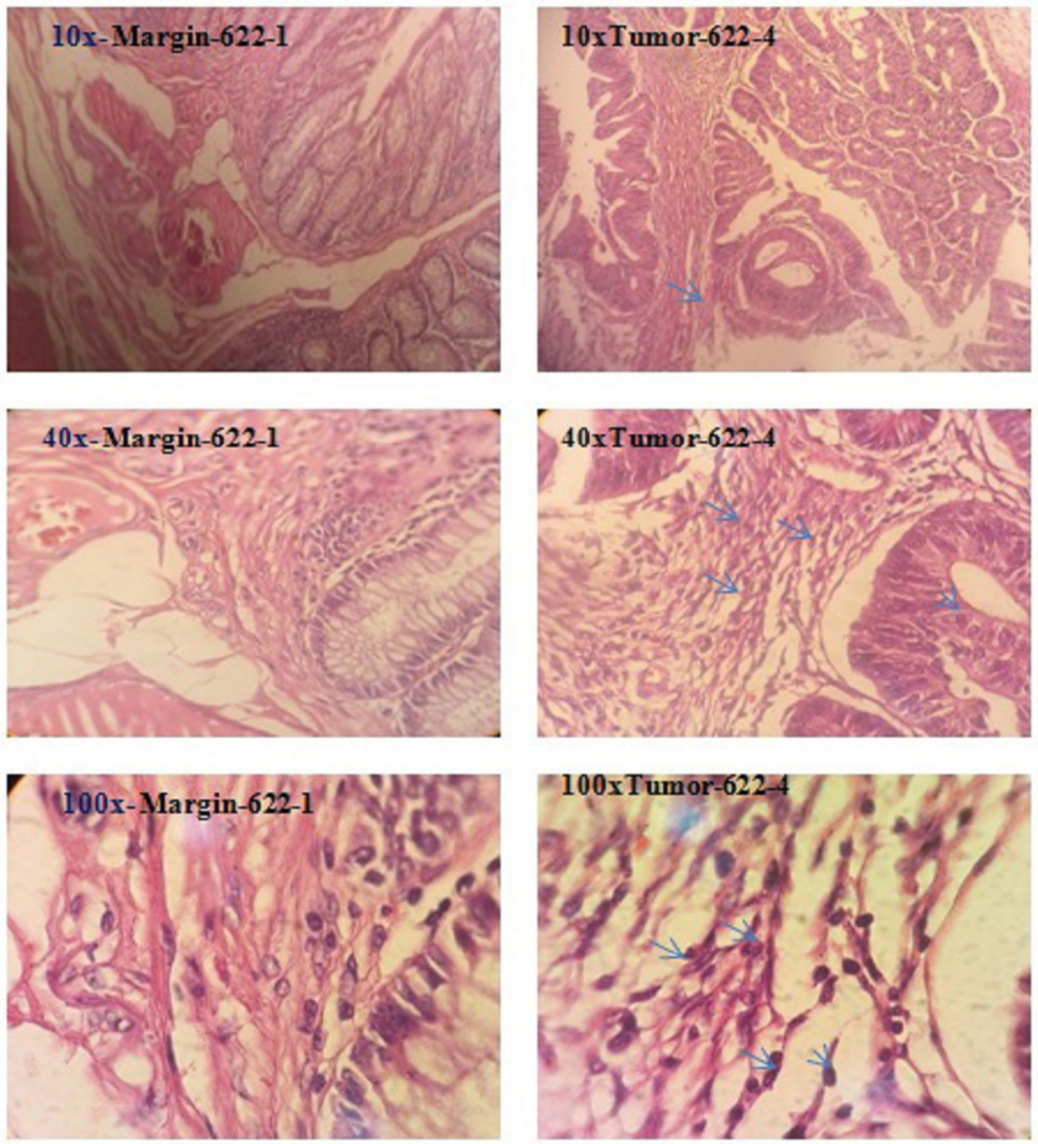
Microscopic assessment of tumor and margin samples of a stained tissue section on a glass microscope slide with various magnifications Arrows represented lymphocyte rich effusion and pleomorphic nuclei with high N/C ratio creating glandular structure associated with desmoplastic stroma. N/C ratio: nucleus-cytoplasm ratio

Extracted RNA from tissue samples (Figure 2) had concentrations ranging from 20 ng to 50 ng. The accuracy of the PCR with synthesized cDNAs was determined by electrophoresis by evaluating the size of the various fragments in 14 tumor and 16 non-tumor samples.

**Figure 3:**
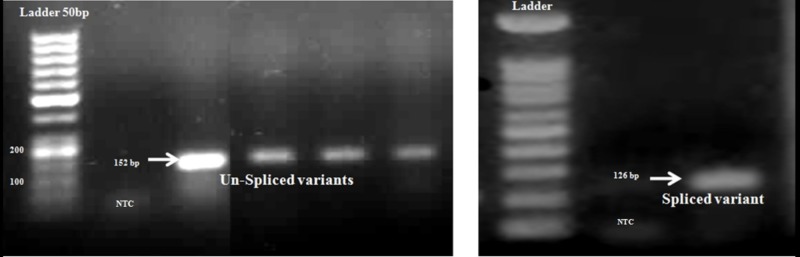
Electrophoresis results of 152 bp and 126 bp fragments associated with spliced and non-spliced variants of tumor and non-tumor samples, on 1.3% agarose gel NTC, negative control; bp: base pair

Figure 3 shows the products associated with non-spliced and spliced variants of 152 bp and 126 bp, respectively, using the primers in Table 1.

**Figure 4:**
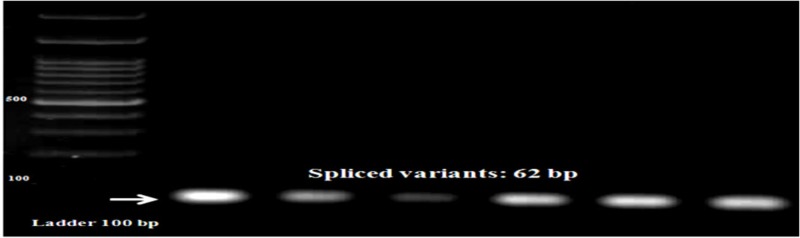
62 bp products associated with spliced variants of the studied samples on 1.3% agarose gel NTC: negative control; bp: base pair

Additionally, we used another set of primers to amplify the spliced variant of XBP again. The 62 bp band confirmed the spliced products (Figure 4). Also, the results of sequencing were evaluated with basic local alignment search tool (BLAST), molecular evolutionary genetics analysis 4.0 (MEGA 4.0) and Chromas software.

**Figure 5:**
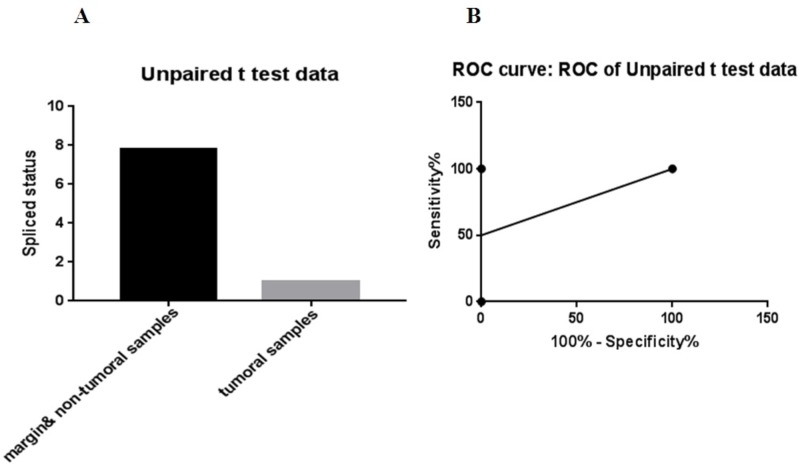
A: Comparison of the number of tumor samples that XBP gene spliced opposed to non-tumor samples, B: ROC analysis of this comparison (cut off<4.4; 95% CI, 2.5% to 100%, specificity and sensitivity: 100%) using GraphPad Prism software version 7.0. ROC: receiver operating characteristic; CI: confidence interval; XBP: X-box binding protein

The data showed there were more than 7.8 times the total number of spliced variants in the marginal tumor samples than in the tumor tissue samples (P<.05) (Figure 5).

## Discussion

According to statistical information from the Center for Disease Control and Prevention in the United States and the United Kingdom, CRC is the leading cause of cancer death after lung cancer [1-2]. In studies on the signaling pathways involved in CRC and molecular research of clinical samples in patients with cancer, multiple pathways, including response pathways to ER stress, undergo changes [3]. ER stress is caused by the accumulation of unfolded or inappropriately folding proteins. Upon the activation of the pathway responding to ER stress, several transcription factors that promote apoptosis are activated. Changes in expression of genes in this pathway, including HSPA5 as a marker of activation of this pathway, are related to a variety of cancers including breast, gastric, lung, colon and prostate cancers characterized by high pathologic levels, tumor recurrence, and low survival [3,7,11]. The location of the XBP gene is 22q12.1, although a pseudogene of XPB has also been identified on chromosome 5. This gene encodes a transcription factor containing the basic leucine-zipper (bZIP) domain that connects to the X-Box and, as such, is called the X-Box Binding Protein. XBP is essential for the survival of a tumor under conditions of oxygen deficiency; for example, it regulates hypoxia-inducing factor (HIF) 1α involved in the advancement of tumorigenicity in triple negative breast cancer [12]. In the process of activating the response pathway to ER stress, the mRNA of this gene undergoes unusual splicing by the gene IRE1 [13].

**Figure 6:**
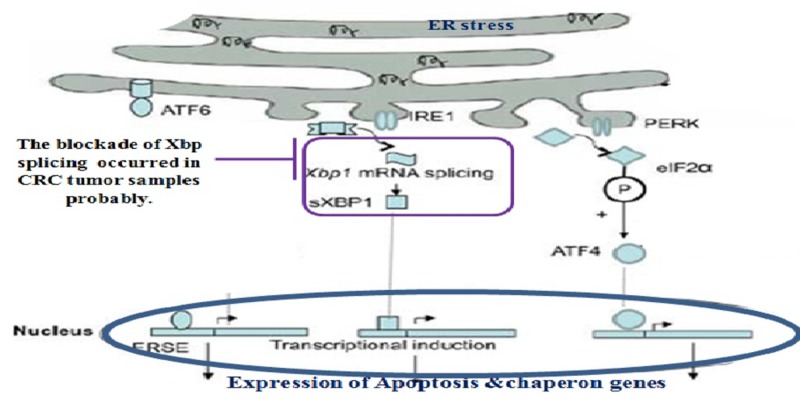
The results of our study showed that with the block of XBP splicing in tumor samples, apoptosis is probably inhibited downstream of the endoplasmic reticulum stress response pathway XBP, X-box binding protein; mRNA, messenger RNA; CRC, colorectal cancer

A 2012 study found that inhibition of XBP splicing can be considered one of the treatments strategies for melanoma [10]. Considering the paradoxical role of the ER stress response pathway in different cancers and its undefined mechanisms in CRC, we chose to study XBP splicing in tumor and marginal CRC tissue samples by a simple in-house PCR evaluation. Our data showed that the spliced variant of XBP in non-tumor and tumor margin tissues were higher than that in the tumor tissue samples of CRC. A schematic view of the activation of the ER stress response pathway and the activation of apoptosis is represented in Figure 6 [14]. The results of our study may logically indicate that, in the tissue context of colorectal tumors, the apoptotic process downstream of the ER stress response pathway was not activated by non-splicing of XBP (Figure 6).

Considering the role of microRNAs (miRNAs) as regulators of signaling pathways in various types of cancer [15], it is suggested that miRNAs affecting the components of this signaling pathway, including XBP, should be further assessed. Our sample size is small, so it is necessary to check more clinical tissue samples and expressions of other genes involved in this signal transduction pathway.

## Conclusions

We found an inverse relationship between XBP transcription and CRC tumor tissue, possibly due to the inactivation of apoptosis in the downstream response to ER stress. However, the remaining genes in this pathway should undergo gene expression analysis using a greater number of samples.
